# Spinal Botulinum Neurotoxin B: Effects on Afferent Transmitter Release and Nociceptive Processing

**DOI:** 10.1371/journal.pone.0019126

**Published:** 2011-04-29

**Authors:** Polly P. Huang, Imran Khan, Mohammed S. A. Suhail, Shelle Malkmus, Tony L. Yaksh

**Affiliations:** 1 Department of Biological Sciences, University of California San Diego, La Jolla, California, United States of America; 2 United States Food and Drug Administration, Department of Health and Human Services, Bethesda, Maryland, United States of America; 3 School of Medicine, University of California San Diego, La Jolla, California, United States of America; 4 Department of Anesthesiology, University of California San Diego, La Jolla, California, United States of America; University of Cincinnatti, United States of America

## Abstract

Botulinum neurotoxin B (BoNT-B) mediates proteolytic cleavage of VAMP I/II (synaptobrevins I/II), which prevents vesicle-membrane fusion and blocks neurotransmitter release. In the present study, we investigated the effects of BoNT-B on neurotransmitter release *in vivo* from spinal primary afferent sensory fibers and the effects of this blockade on nociception. With intrathecally (IT) delivered BoNT-B in C57B/l6 mice, we characterized the effects of such block on the release of substance P (SP) from spinal afferent nociceptors (as measured by neurokinin-1 receptor, NK1-R, internalization), spinal neuronal activation (as indicated by spinal C-Fos expression) and nociceptive behavior after intraplantar (IPLT) formalin. In addition, we investigated the effect of IT BoNT-B on spinal nerve ligation-induced tactile allodynia. A single percutaneous IT injection of BoNT-B 0.5 U at 2 or 5 days prior to IPLT formalin reduced NK1-R internalization and C-Fos expression. These effects correlated with BoNT-B cleavage of VAMPI/II protein in tissue lysate. IT BoNT-B also produced a corresponding reduction in phase 2 of formalin-evoked flinching behavior for over 30 days after IT injection. In mice with spinal nerve ligation (SNL), tactile allodynia was observed, which was attenuated by IT BoNT-B 0.5 U over the next 15 days, as compared to vehicle animals. These effects were observed without effects upon motor function. The specificity of the IT BoNT-B effect is indicated by: i) IT co-injection of BoNT-B and anti-BoNTB antibody prevented effects on SP release, and ii) IT BoNT-B 50 U in the Sprague Dawley rats showed no effect on formalin-evoked flinching or SNL-induced tactile allodynia, which is consistent with rat resistance to BoNT-B. IT BoNT-B blocks transmitter release from spinal primary afferents, and attenuates inflammatory nociceptive response and spinal nerve injury-induced neuropathic pain, in the absence of motor impairment. These observations provide an initial assessment of the ability of IT BoNT-B to regulate spinal nociceptive processing.

## Introduction

Botulinum neurotoxins (BoNTs) are metalloproteases produced by *Clostridium botulinum*. They mediate proteolytic cleavage of protein subunits in the family of soluble N-methylaleimide-sensitive attachment protein receptor (SNARE) proteins in the synaptic terminal. There are seven BoNT serotypes (A–G). Each neurotoxin serotype specifically and non-competitively cleaves one site of a member of the SNARE protein complex: botulinum neurotoxin serotype A (BoNT-A) cleaves SNAP-25. Botulinum neurotoxin serotype C (BoNT-C) cleaves syntaxin. Botulinum neurotoxin serotypes B, F, and G cleave VAMP/synaptobrevin protein [1]. Such enzymatic cleavage results in inhibition of vesicle-membrane fusion and prevents the release of neurotransmitters. Previous studies have shown effects of BoNTs on acetylcholine release from motor neurons and effects on muscle. SNARE proteins, however, play a ubiquitous role in vesicle-membrane fusion during neurotransmitter release in both the peripheral and central nervous systems. Several studies have demonstrated that treatment of neuronal systems and cell cultures that BoNT-A inhibits release of acetylcholine, substance P [2], glutamate [3], and calcitonin gene-related peptide [4], [5]. Despite the widespread role of SNARE proteins in CNS and PNS, there has been little work on the effects of BoNTs on neurotransmitter release in systems other than neuromuscular junction. Furthermore, the majority of this work has focused on *ex vivo* systems and the effects on behavior have been described in a limited fashion.

Accordingly, in this series of studies, we examined the effects of the intrathecal (IT) delivery of BoNT-B on substance-P (SP) release from spinal primary afferent C-fibers in the spinal cord *in vivo* upon stimulation by intraplantar (IPLT) formalin in the hind paw. SP-specific binding to neurokinin-1 receptors (NK1-R) in the superficial spinal dorsal horn, where C-fibers terminate, induces NK1-R internalization which can be visualized immunohistochemically as a quantitative assay for neurotransmitter release. IT BoNT-B effects on post-synaptic activation was also demonstrated by spinal C-Fos protein expression. In order to demonstrate the functional significance of BoNT-B effects, we examined its effects upon IPLT formalin-induced flinching behavior and spinal nerve ligation-induced tactile allodynia. These studies revealed a robust effect of IT BoNT-B on neurotransmitter release and spinal C-Fos expression, which correlated with suppression of formalin-evoked pain behavior and nerve injury-induced hyperalgesia. The specificity of this effect is supported by the observation that intrathecal BoNT-B failed to have any effect after delivery in rats, a finding consistent with a single amino acid variation in rat VAMPI protein rendering resistance to BoNT-B cleavaging activity.

## Methods

### Ethics Statement

All studies undertaken in this study were carried out according to protocols approved by the Institutional Animal Care and Use Committee of the University of California, San Diego.

### Animals

Adult male C57B/l6 mice and Sprague Dawley rats (Harlan Sprague Dawley Inc., Indianapolis, IN). Animals were housed in vivarium minimum of 2 days before use, maintained on a 12/12 hour day-night cycle, and are given food and water *ad libitum*.

### Drug Delivery

#### Mouse intrathecal injections

Animals were anesthetized using 5% isoflurane for induction and 3% isoflurane during the IT injection. A 30G needle was inserted into the intrathecal space at L5–L6, and a tail flick was observed as indication of correct placement of the injection. Drugs were delivered using a Hamilton syringe. Intrathecal injections of NaCl 0.9% or drug were given in volumes of 5 µL for each dose given, unless otherwise stated.

#### Rat intrathecal injections

While anesthetized using 5% isoflurane, rats were prepared with lumbar intrathecal (IT) catheters as previously described [6], [7]. After 5–7 days recovery, rats were entered into the study to receive drug in volume of 10 µL, unless otherwise stated. Each injection is followed by 10 µL of saline flush.

### Drugs

Botulinum neurotoxin B (BoNT-B; Myobloc®, Solstice Neurosciences Inc., South San Francisco, CA) was provided in solution containing 5000 U/mL. BoNT-B was diluted in NaCl 0.9% such that the dose to be delivered was present in a volume of 5 µL for mouse studies and 10 µL for rat studies, unless otherwise stated. In both rat and mouse studies, intraplantar formalin (5%, 20 µL) was given in the left hind paw. For IT injections of BoNT-B titer, BoNT-B was incubated with anti-BoNT-B antibody (rabbit polyclonal; Abcam Inc., San Francisco, CA) at effective binding concentrations determined in titer assay for 1 hour at 37°C prior to IT injection.

### Morbidity and Motor Function

#### Morbidity

Mice received intrathecal injection of BoNT-B at doses 0.1 U–50 U. Clinical observations were made for indications of toxicity: piloerection, labored breathing, normal grooming behavior, normal ambulation, and exploratory behavior. In order to assess weight loss, mice that received maximal tolerable dose of BoNT-B were weighed 7 days after intrathecal injection.

#### Grip strength

Five days after receiving IT NaCl 0.9% or BoNT-B 0.5 U, animals were placed on top of a wire mesh mounted on a scale. The experimenter lifts the animal from the wire mesh by the tail. The grip strength is recorded as the maximal force at the point of release from the wire mesh by the hind paws.

#### Motor function

Animals were required to remain suspended underneath a wire mesh for up to 1 minute. When placed upside-down under the wire mesh, animals were required to grip onto the wire grid with front and hind paws, and grip strength is required to support their body weight. Normal exploratory movement while suspended underneath the wire mesh was also observed, as indication of exploratory behavior and motor coordination.

### Nociception

#### Formalin-Induced Flinching

A metal band was placed on the left hind paw of the mouse and acclimated in the behavior testing chamber at least 1 hour prior to formalin injection. Intraplantar (IPLT) injection of formalin (5%, 20 µL) was administered in the marked hind paw and the animal was immediately returned to the detection device. Flinching behavior was collected using an automated system for 40 minutes following IPLT formalin injection [8].

#### Spinal Nerve Ligation (SNL)

Same spinal nerve ligation procedure was used for rats and mice following procedures described in Kim and Chung, 1992 [9]. Animals were anesthetized using xylazine (10 mg/kg) and ketamine (100 mg/kg) cocktail and 3% isoflurane during ligation surgery. The L6 transverse process is removed to expose the L5 and L6 nerve roots. A 6-0 silk suture is tied on the L5 spinal nerve distal to the spinal cord. Animals were given 1 mL lactated Ringer's solution with carprofen subcutaneously immediately after surgery. At 5–7 days after ligation surgery, increase in tactile hypersensitivity was observed (tactile allodynia).

#### Tactile Allodynia Assessment

Animals were allowed to acclimate in clear plastic testing chambers on wire mesh for at least 1 hour prior to testing. To assess paw withdrawal threshold (PWT), von Frey filaments (Touch Test® Sensory Evaluators) were applied to the left hind paw which is ipsilateral to the site of spinal nerve ligation. Using the up-down method of von Frey filament application [10], six readings were taken for statistical analysis of tactile threshold.

### Spinal Cord Harvest

For NK1-R internalization and spinal C-Fos expression studies, animals were sacrificed at 10 minutes and 2 hours, respectively, after IPLT formalin using intraperitoneal (IP) Euthosol (200 µL). Animals were intracardially perfused with 0.9% NaCl, and fixed using 4% paraformaldehyde. Laminectomy was performed to extract the spinal cord. Harvested spinal cords were incubated for 24 hours in 4% paraformaldehyde, followed by 20% sucrose and 30% sucrose. For Western blot assays for VAMP I/II cleavage, animals were anesthetized using 5% isofluorane and sacrificed by decapitation. Spinal cord was harvested by hydroextrusion. Spinal cord was placed in lysis buffer (0.5% Triton X-100, 50 mM Tris, 150 mM NaCl, and nanopure H_2_O), with protease and phosphatase inhibitors (Sigma Aldrich Co., St. Louis, MO), and homogenized by sonication.

### Immunohistochemistry

#### Tissue preparation

For NK1-R internalization studies, lumbar tissue samples of L2–L5 incubated in rabbit anti-NK1-R (Advanced Targeting Systems) and mouse anti-NeuN (Chemicon) antibodies; followed by 2° Antibodies Fluroscein (FITC)-conjugated AffiniPure F(ab)2 Fragment Donkey Anti-Rabbit IgG and Rhodamine Red™-X-conjugated AffiniPure Donkey Anit-Mouse IgG (JacksonImmuno Research). For C-Fos expression studies, lumbar tissue samples of L2–L5 were incubated in anti-C-Fos (CalBiochem) and biotin-conjugated anti-NeuN (Chemicon) primary antibodies. Tissue was then incubated in fluorescent conjugated 2° antibodies: Streptavidin-AlexaFluor® 555 conjugate and AlexaFluor® 488 goat anti-rabbit IgG (Molecular Probes).

#### Tissue analysis

Dorsal horn regions of antibody-stained spinal cord tissue were scanned using confocal microscopy (Leica TCS SP5) 63× oil immersion objective. Images were viewed on Adobe Photoshop CS2®. NK1-R internalization is characterized based on a set of criteria: i) formation of an outline of NK1-R immunoreactivity around NeuN immunoreactivity, ii) distortion of NK1-R outline and appearance of endosomes indicative of NK1-R internalization. The total number of NK1-R immunoreactive neurons, with and without NK1-R internalization, was counted and used to calculate percentage of cells showing NK1-R internalization. Counting was performed without knowledge of treatment.

### Stastistical Analysis

Statistical analysis was performed on GraphPad Prism (GraphPad Software, Inc.) using two-way ANOVA parametric analsysis. Results are expressed as means ± SEM. P<0.05 was taken as the level of significance, unless otherwise stated. All behavioral data are presented as the mean ± SEM of the respective data set plotted versus time. For Western blot quantification, blot was developed on film and photo scanned. Band intensity was analyzed usin ImageQuant TL software (GE Lifesciences Inc.).

All behavioral data are presented as the mean ±SE of the respective data set plotted versus time. For the formalin model, the area under the curve (flinch count vs. time) was calculated for phase 1 (0–10 minutes) and phase 2 (11–40 minutes). For the tactile allodynia data in the nerve injury model, paw withdrawal thresholds (PWT) were plotted as percent of the pre-BoNT-B treatment baseline PWT for each animal to reduce variations between animals in the analysis. The effect-over-time curves for each group was calculated and presented in the tactile allodynia data accordingly.

## Results

### Dose tolerability of BoNT-B in the mouse

**Figure 1:**
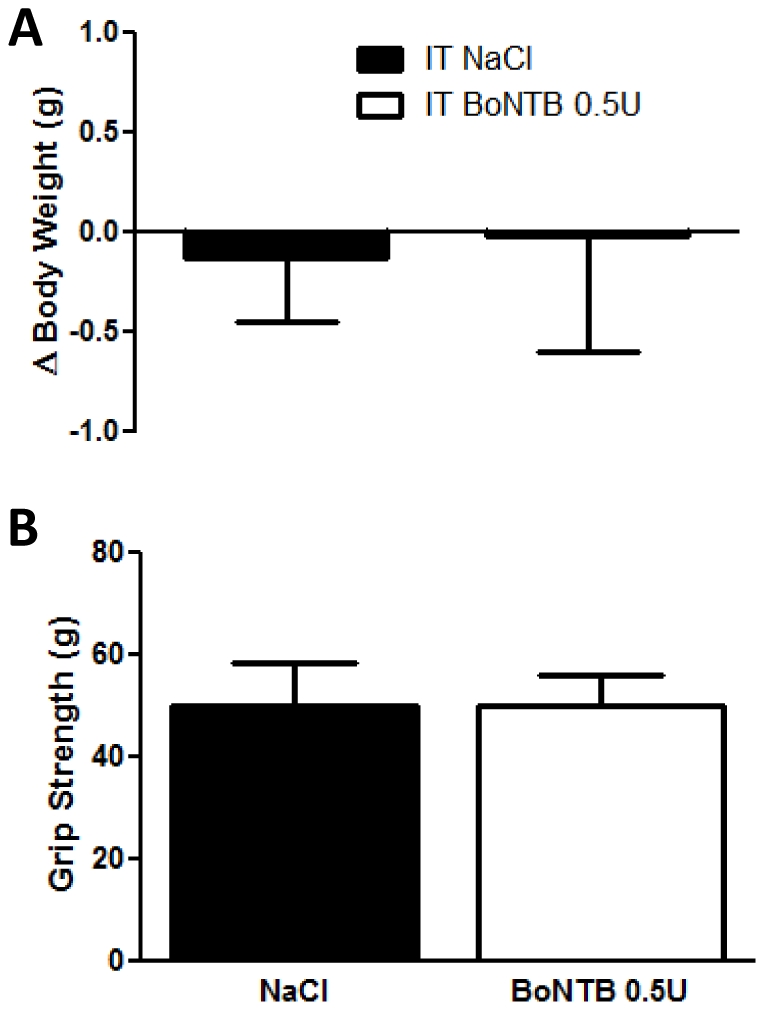
BoNT-B effect on motor function and toxicity in the mouse. A) Grip strength of mice that received IT NaCl (vehicle, n = 5) or IT BoNTB 0.5 U (n = 5) 5 days prior to measurement on top of a wire mesh mounted on a weigh scale. Grip strength was recorded as the maximum force at the point of release from the wire mesh. B) Histogram shows change in body weight of mice that received IT NaCl (vehicle, n = 4) and IT BoNTB 0.5 U (n = 6) measured 7 days after IT injection. Bars represent mean ± SD.

In order to determine a maximal tolerable dose of intrathecally delivered BoNT-B in the mouse, animals received intrathecal (IT) injection of BoNT-B, were observed for clinical indications of toxicity and were tested for motor impairment and grip strength. At doses 1 U–50 U given intrathecally, onset of motor disability was observed within 24 hours after the injection. Because of evident motor impairment and clinical indications of toxicity, animals required sacrifice. The maximal tolerable dose was determined to be 0.5 U, with survival and normal motor function observed at 7 days after IT injection (table 1). The same animals showed similar grip strength of the hind paw, as compared to IT NaCl animals at 5 days after IT injection (figure 1A). The same animals were required to hang underneath a wire mesh for up to 1 minute. Animals that received the maximal tolerable dose of BoNT-B showed normal motor coordination, grip strength and exploratory behavior on the wire mesh. Both NaCl and BoNT-B treated animals remained suspended upside down underneath the wire mesh for the required time (data not shown). In addition, weight loss was monitored as one clinical indication for toxicity. Animals that received BoNT-B 0.5 U also did not show significant weight loss at 7 days after intrathecal injection, as compared to IT saline pre-treated animals (figure 1B). IT BoNT-B 0.5 U was established as the maximal tolerable dose for experiments from this point forward.

**Table 1:** Morbidity after IT BoNT-B injection.

BoNT-B Doses	Route	Survival (days)	N	Clinical Obs*
50 U/10 uL	IT	1	3	moribund
5 U/5 uL	IT	1	2	moribund
1 U/5 uL	IT	1	2	motor impairment
0.5 U/5 uL	IT	>2	2	normal
0.1 U/5 uL	IT	>2	2	normal
0.5 U/5 uL	IT	>7	4	normal
0.1 U/5 uL	IT	>7	4	normal

Table shows summary of clinical observations following intrathecal injection of BoNT-B in mouse. Maximal tolerable dose was determined to be BoNT-B 0.5 U/5 µL.

*Clinical observation was made at time of sacrifice.

### BoNT-B cleavage of spinal VAMP I/II

**Figure 2:**
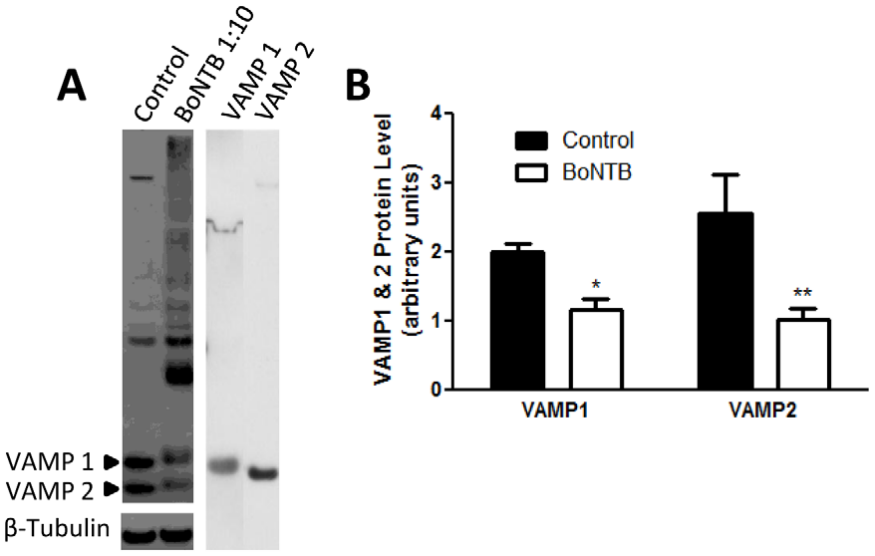
BoNT-B cleavage of spinal VAMP I/II. A) Immunoblots show VAMP I/II protein of control lumbar spinal cord tissue lysate and tissue lysate incubated with BoNT-B (1∶10 concentration). VAMP I and II bands are confirmed by VAMP I (19 kDa) and VAMP II (17 kDa) synthetic peptides. B) Quantification of band intensity for control and BoNTB treated tissue VAMP I and II compared to β-Tubulin.

In order to determine the cleavage of spinal VAMP I/II by active BoNT-B, spinal cord tissue lysate was incubated with BoNT-B. Western blot of control spinal cord tissue lysate shows both VAMP I (19 kDa) and VAMP II (17 kDa) proteins. Tissue lysate incubated with BoNT-B show significant decrease in VAMP I/II whole protein (figure 2). VAMP I/II cleavage product of smaller molecular weight was not observed, most likely due to rapid enzymatic degradation.

### Characterization of NK1-R expression and internalization

**Figure 3:**
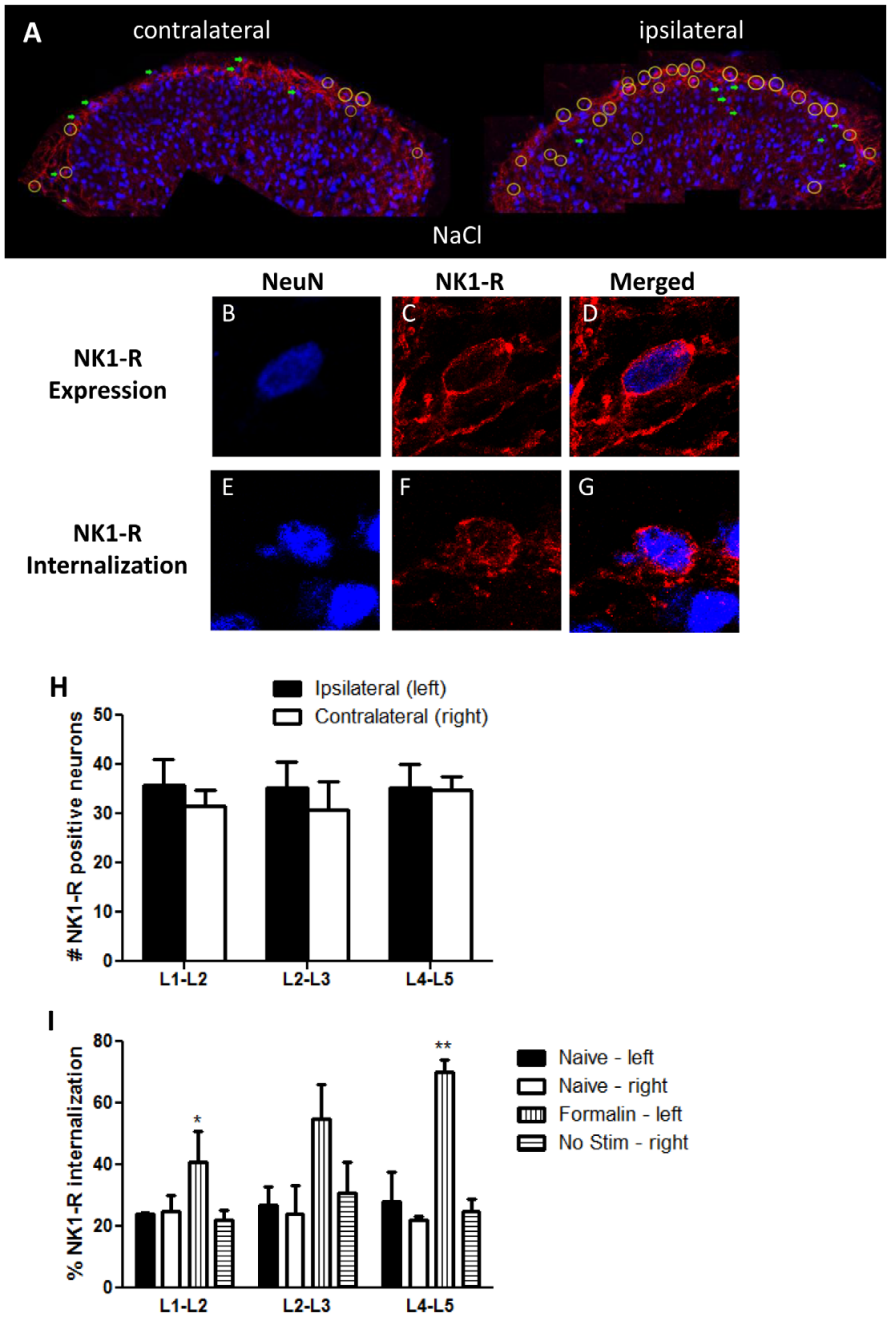
Characterization of NK1-R expression and internalization in the spinal dorsal horn. Spinal dorsal horn NK1-R internalization was uniformly counted for expression and internalization after IPLT formalin injection. A) Representative confocal image of mouse dorsal horns ipsilateral and contralateral to the site of formalin injection showing location of NK1-R positive cells (with, yellow circles; without, green arrows) internalization. B–D) Examples of neuron in the dorsal horn showing expression of neurokinin-1 receptors. NK1-R expression is characterized by co-localization of NeuN and NK1-R immunoreactivity. Merged image shows outlined of NK1-R on the membrane. E–G) Neuron showing NK1-R internalization, characterized by co-localization of NeuN and NK1-R, as well as distortion of membrane outline marked by NK1-R immunoreactivity and formation of endosomes indicative of receptor internalization. H) Graph showing number of neurons showing NK1-R expression in spinal dorsal horn along the lumbar segment. NK1-R expression is shown to be consistent along the lumbar spinal cord. I) Graph shows NK1-R internalization along the lumbar segment at 10 minutes after IPLT formalin in the left hind paw (all n = 3). Bars represent mean ± SEM; * and ** P value<0.5.

Substance P specifically binds to neurokinin-1 receptors (NK1-R) in the superficial dorsal horn (laminae I and II), causing receptor internalization. Quantification of NK1-R internalization in the superficial dorsal horn was used as an assay for neurotransmitter release. To quantify NK1-R internalization, we first standardized characterization of NK1-R expression and internalization at high magnification (63×). In confocal images of lumbar tissue stained for NeuN and NK1-R immunoreactivity (figure 3B–G), NK1-R expression is characterized by appearance of an outline of NK1-R immunoreactivity surrounding NeuN immunoreactivity. NK1-R internalization is characterized by distortion of the NK1-R outline and appearance of endosomes. This indicates migration of NK1-R immunoreactivity as receptors undergo internalization. Confocal images of mouse lumbar dorsal horns are counted for NK1-R expression and internalization at high magnification based on these criteria. In animal that received IT NaCl prior to IPLT formalin, ipsilateral dorsal horn showed significantly elevated NK1-R internalization, as compared to contralateral dorsal horn to the site of formalin injection (Figure 3A).

We then characterized NK1-R expression in the superficial dorsal horn rostrocaudally along the lumbar segment. Confocal images from L1–L5 show statistically similar numbers of neurons positive for NeuN immunoreactivity and NK-R expression (approximately 30%) in the superficial dorsal horn (figure 3H). We next examined lumbar segments L1–L5 for maximal formalin-induced NK1-R internalization. Naïve, unstimulated mice showed minimal (approximately 20–30%) basal NK1-R internalization in the left and right dorsal horns. This was established as constitutive NK1-R internalization in the absence of stimulation. Representative confocal images from animals that received IPLT formalin show increasing NK1-R internalization from L1 through L5. NK1-R internalization in the ipsilateral dorsal horn was maximal in L4–L5 at 10 minutes after formalin injection (figure 3I). In subsequent studies of formalin-evoked NK1-R internalization, representative confocal images from L4–L5 were counted for NK1-R expression and NK1-R internalization.

### Effects of IT BoNT-B on formalin-evoked neurotransmitter release

**Figure 4:**
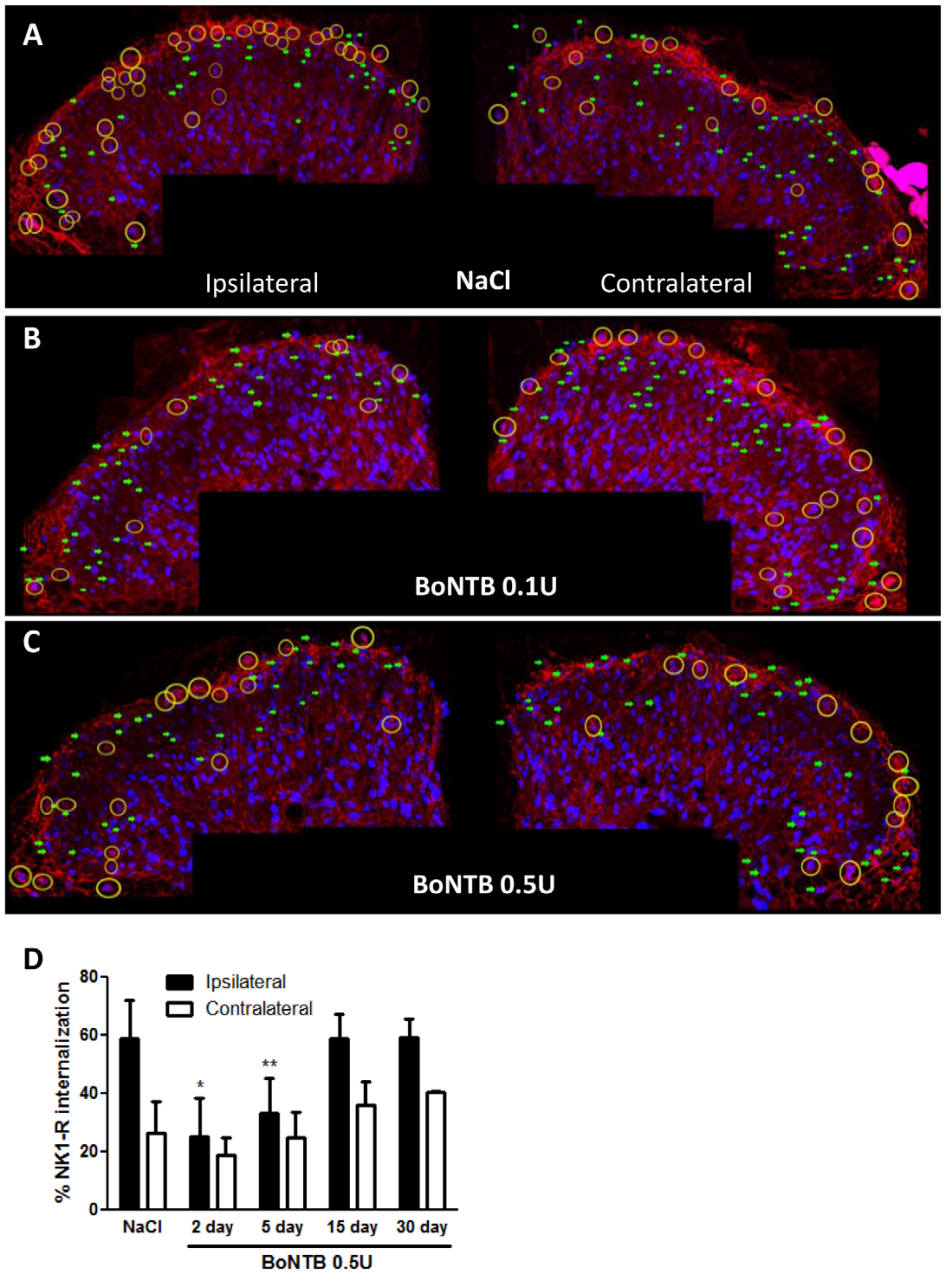
IT BoNT-B on neurotransmitter release from spinal primary afferent C-fibers. A–C) Figure shows representative confocal images of dorsal horns of IT BoNTB pretreated and vehicle animals. Mice received IT injections 5 days prior to IPLT formalin. Both the ipsilateral and contralateral dorsal horns are counted for NK1-R expression (green arrows), and for NK1-R internalization (yellow circles). At 10 mins after IPLT formalin IT NaCl pretreated animal shows significant increase in NK1-R internalization in the dorsal horn ipsilateral to the site of formalin injection. In contrast, in the IT BoNTB 0.1 U (n = 4) and BoNTB 0.5 U (n = 4) pre-treated animal, ipsilateral formalin-induced NK1-R internalization is significantly reduced, as compared to the vehicle animals (n = 4). There was no significant difference in contralateral NK1-R internalization between the BoNTB pretreated and vehicle animals. D) Percentage of NK1-R expressing neurons in the dorsal horn that show internalization after IPLT formalin. Ipsilateral dorsal horn shows marked increase in NK1-R internalization in the vehicle animals (n = 5, pooled) after IPLT formalin. Animals that received IT BoNTB 0.5 U pretreatment 2 days (n = 5) and 5 days (n = 4) prior to formalin showed marked reduction in NK1-R internalization after IPLT formalin in the hind paw. At 15 days (n = 3) and 30 days (n = 3) after IT administration, BoNTB 0.5 U did not show significant effect on formalin-evoked NK1-R internalization, as compared to vehicle animals. Both vehicle and BoNTB pre-treated animals showed similar NK1-R internalization in the contralateral dorsal horn. Bars represent mean ± SEM; * and ** P value<0.5.

The effects of spinal BoNT-B on primary afferent substance-P release from C-fibers were characterized by examining the effects of intrathecally delivered BoNT-B on the release of SP evoked by the unilateral intraplantar delivery of formalin. Animals received IT NaCl or BoNT-B (0.1 U–0.5 U), at intervals prior to IPLT formalin in the left hind paw. In animals that received intrathecal (IT) NaCl pretreatment, dorsal horn ipsilateral to the site of formalin injection shows marked increase in NK1-R internalization, approximately 53%, as compared to the contralateral unstimulated dorsal horn, approximately 25% (figure 4A). In contrast, animals that received IT BoNT-B 0.5 U pretreatment showed a marked reduction in the incidence of formalin-evoked NK1-R interanlization in the ipsilateral dorsal horn (approximately 25%) as compared to the ipsilateral dorsal horn of a vehicle animal (figure 4B–C). NK1-R internalization in the contralateral dorsal horns of IT BoNT-B and NaCl pretreated animals did not show any difference after IPLT formalin. Two-day and 5-day IT BoNT-B pretreatment showed comparable reduction in NK1-R internalization (figure 4D), which suggests a sustained effect of BoNT-B on primary afferent neurotransmitter release. In separate groups of animals, NK1-R internalization at 15 days and 30 days after IT treatment was comparable to IT NaCl pretreated animals, indicating recovery from IT BoNT-B effects on neurotransmitter release between 5 days and 15 days after IT treatment.

**Figure 5:**
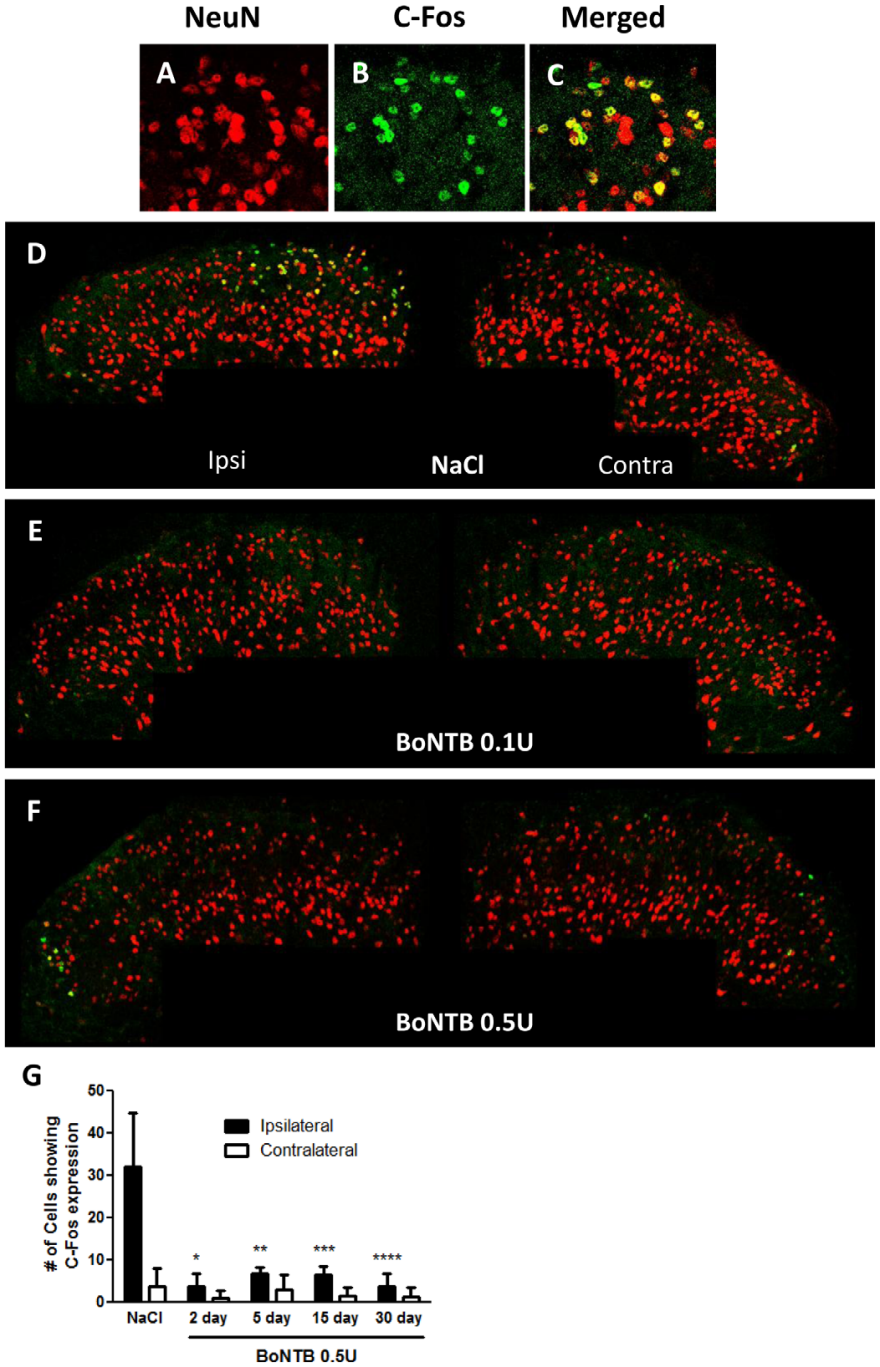
IT BoNT-B reduces IPLT formalin-evoked C-Fos activation. A–C) Co-localization of NeuN and C-Fos immunoreactivity indicating C-Fos expression at 2 hrs after IPLT formalin. D–F) Confocal images representative of C-Fos expression after IPLT formalin. In animals that received IT NaCl 2 days prior to formalin, IPLT formalin induced marked increase of C-Fos expression in ipsilateral dorsal horn interneurons. In contrast, mice that received IT 2-day pretreatment of either BoNTB 0.1 U (n = 4) or 0.5 U (n = 5) showed significant reduction in C-Fos protein expression, as compared to IT NaCl pretreated animals (n = 5). G) Quantification of C-Fos protein expression in the superficial dorsal horn at 2 hours after IPLT formalin. In different groups of animals, pretreatment of IT BoNTB 0.5 U 2 days (n = 5), 5 days (n = 3), 15 days (n = 2), and 30 days (n = 3) prior to IPLT formalin show significant decrease in formalin-evoked C-Fos expression in the ipsilateral dorsal horn, as compared to vehicle animals (n = 5). Bars represent mean ± SEM; P-value<0.5.

Based on IT BoNT-B effects on formalin-evoked neurotransmitter release from primary afferent sensory neurons, we next investigated whether IT BoNT-B pre-treatment would result in suppression of non-afferent neuron activation after IPLT formalin. C-Fos protein expression, which is an immediate early gene expressed upon neuronal activation, is used in our study as a marker for central neuronal activation. Confocal images of spinal cord tissue showed C-Fos protein activation in the spinal dorsal horn, characterized by co-localization of C-Fos immunoreactivity and NeuN immunoreactivity (figure 5A–C). At 2 hours after IPLT injection, formalin induced elevated C-Fos protein expression in the ipsilateral dorsal horn in vehicle animals that received IT NaCl 2 days prior to formalin (figure 5D). In contrast, animals that received IT BoNT-B 0.1 U and 0.5 U 2-day pretreatment showed marked decrease in formalin-evoked C-Fos expression in the ipsilateral dorsal horn (figure 5E–F), as compared to vehicle animals. This reduction in formalin-induced C-Fos expression was maintained up to 30 days following IT BoNT-B treatment (figure 5G). Confocal images of the unstimulated contralateral dorsal horn did not show statistical difference in C-Fos expression between treatment groups. The data suggest that IT BoNT-B pre-treatment, which blocked neurotransmitter release from primary afferent C-fibers, also reduced activation of superficial dorsal horn interneurons upon stimulation by a painful chemical stimulus.

### Effects of IT BoNT-B on formalin-induced nocifensive behavior

**Figure 6:**
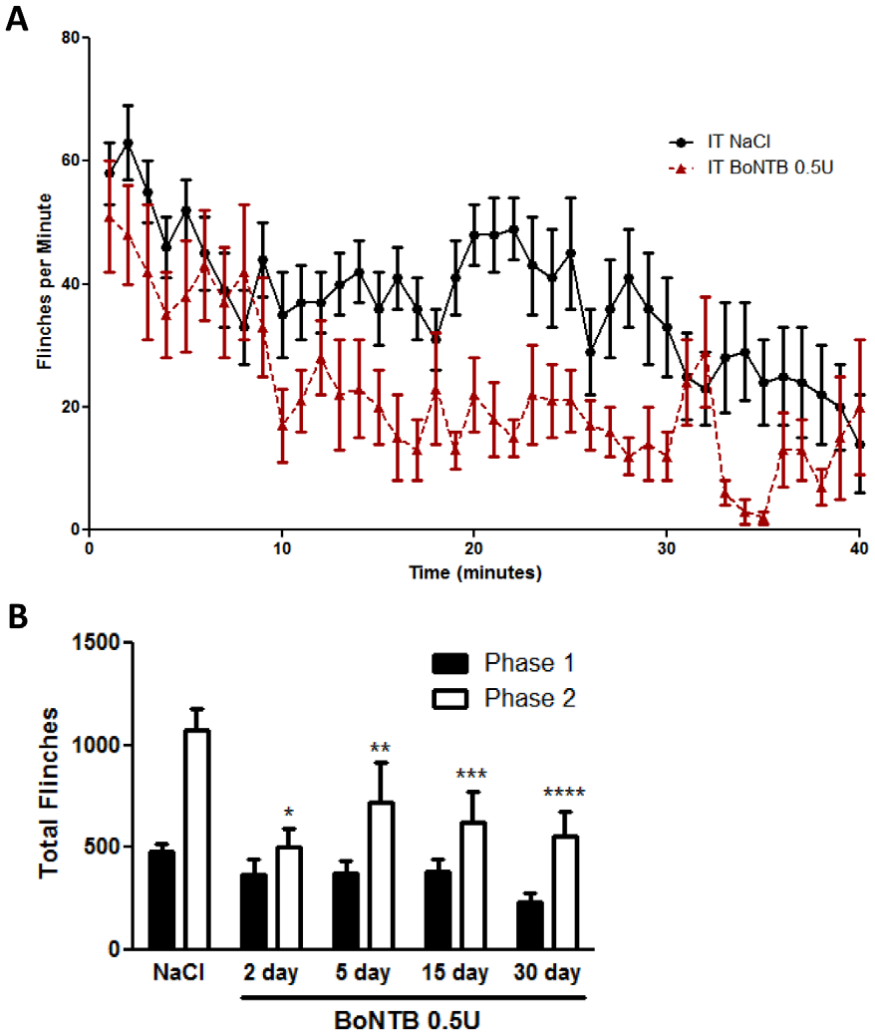
Effects of BoNT-B on formalin-evoked flinching behavior in the mouse. A) Time course of formalin-evoked flinching behavior measured up to 40 mins after formalin. Mice received IT BoNTB 0.1 U (n = 8) or 0.5 U (n = 11) and IT NaCl (n = 12) 2 days prior to IPLT formalin injection. B) Cumulative number of flinches compared between phases. Different groups of mice received IPLT formalin 2 days, 5 days, 15 days, and 30 days after IT BoNTB 0.5 U. Animals that received IT BoNT-B showed reduction of phase 2 of flinching response, compared to NaCl-treated animals. Bars represent mean ± SEM; P-value<0.5.

To determine whether the effects of IT BoNT-B on primary afferents fibers at doses which block neurotransmitter release and C-Fos activation alter nocifensive behavior, the effects of IT BoNT-B on formalin-induced flinching response was examined. Mice received IT NaCl or IT BoNT-B 0.1 U–0.5 U pretreatment 2 days prior to formalin-evoked flinching measurements. IPLT formalin evoked robust biphasic flinching behavior in mice that received IT NaCl pretreatment. This flinching behavior showed a typical time course, suggestive of biphasic response (e.g. phase I: 0–10 minutes and phase II: 11–40 minutes). Mice that received IT BoNT-B 0.5 U 2-day pretreatment showed significant reduction in phase II, but not phase I, of formalin-evoked flinching response, as compared to vehicle animals (figure 6A). Reduction of phase II formalin-evoked flinching behavior was observed in different groups of animals that received IT BoNT-B 5 days, 15 days, and 30 days prior to IPLT formalin (figure 6B). The data indicate that IT BoNT-B pretreatment reduces chemically induced nocifensive behavior in the mouse, and that this reduction in nocifensive behavior is sustained for up to 30 days after a single IT injection.

### Effects of IT BoNT-B on neuropathic pain

**Figure 7:**
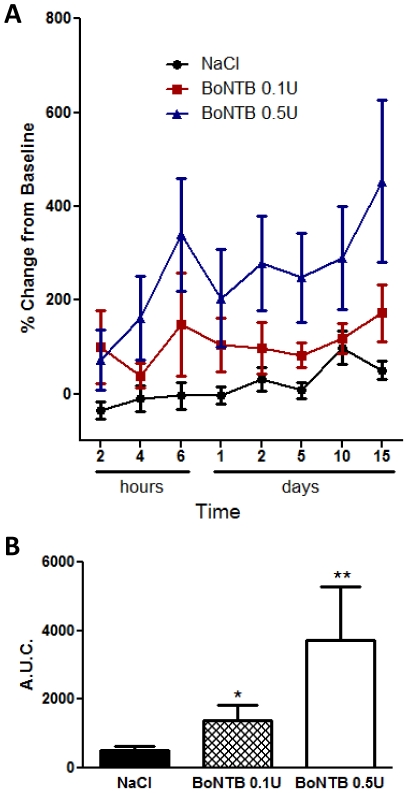
Effects of IT BoNTB on neuropathic hyperalgesia. SNL-induced tactile allodynia after IT BoNT-B. Paw withdrawal threshold (PWT) was measured using von Frey filaments at 14–15 days after SNL surgery and continued up to 15 days after IT injection. A) Time course of tactile allodynia up to 15 days after IT treatment, expressed as % change from baseline. At 14–15 days after SNL surgery, baseline PWT was measured: naïve (n = 8), 1.35±0.53; NaCl, 0.69±0.32; BoNTB 0.1 U, 0.58±0.32; BoNTB 0.5 U, 0.45±0.24. Mice received IT NaCl (n = 8) or BoNTB 0.1 U–0.5 U (n = 6) immediately after baseline PWT was measured at day 14–15 days after SNL (t = 0). B) Area under curve of % change from baseline PWT over 15 days after IT treatment. Bars represent mean ± SEM; * and ** P value<0.5.

Spinal nerve injury induces long-term neuropathic hyperalgesia characterized by long-term tactile allodynia or decreased paw withdrawal threshold (PWT) when stimulated by a normally innocuous mechanical stimulus. Mice that received spinal nerve ligation (SNL) of the L5 spinal nerve show significant decrease in paw withdrawal threshold in response to von Frey filaments, as compared to naïve non-ligated animals by 14–15 days after ligation surgery. Immediately following baseline withdrawal threshold measurements, single IT NaCl or BoNT-B 0.1 U–0.5 U was administered and withdrawal thresholds were assessed up to 15 days after IT injection. Animals that received SNL and IT NaCl maintained paw withdrawal threshold below 1 g for the duration of assessment, with little change from baseline PWT. In contrast, animals that received IT BoNT-B 0.5 U showed increase from baseline PWT within 6 hours after IT treatment. These animals also showed a statistically significant and persistent increase (normalization) in PWT from baseline for the next 15 days after IT treatment (figure 7A–B). The data suggests long-term sustained alleviation of hyperalgesia by IT BoNT-B, as well as long-term dose-tolerability of BoNT-B in the mouse. Although systematic assessments motor function were not undertaken, daily observation of motor behavior revealed that these animals showed normal motor function and symmetrical ambulation, and did not show signs of motor weakness at any time point.

### Antibody binding of BoNT-B and effect on primary afferent neurotransmitter release

**Figure 8:**
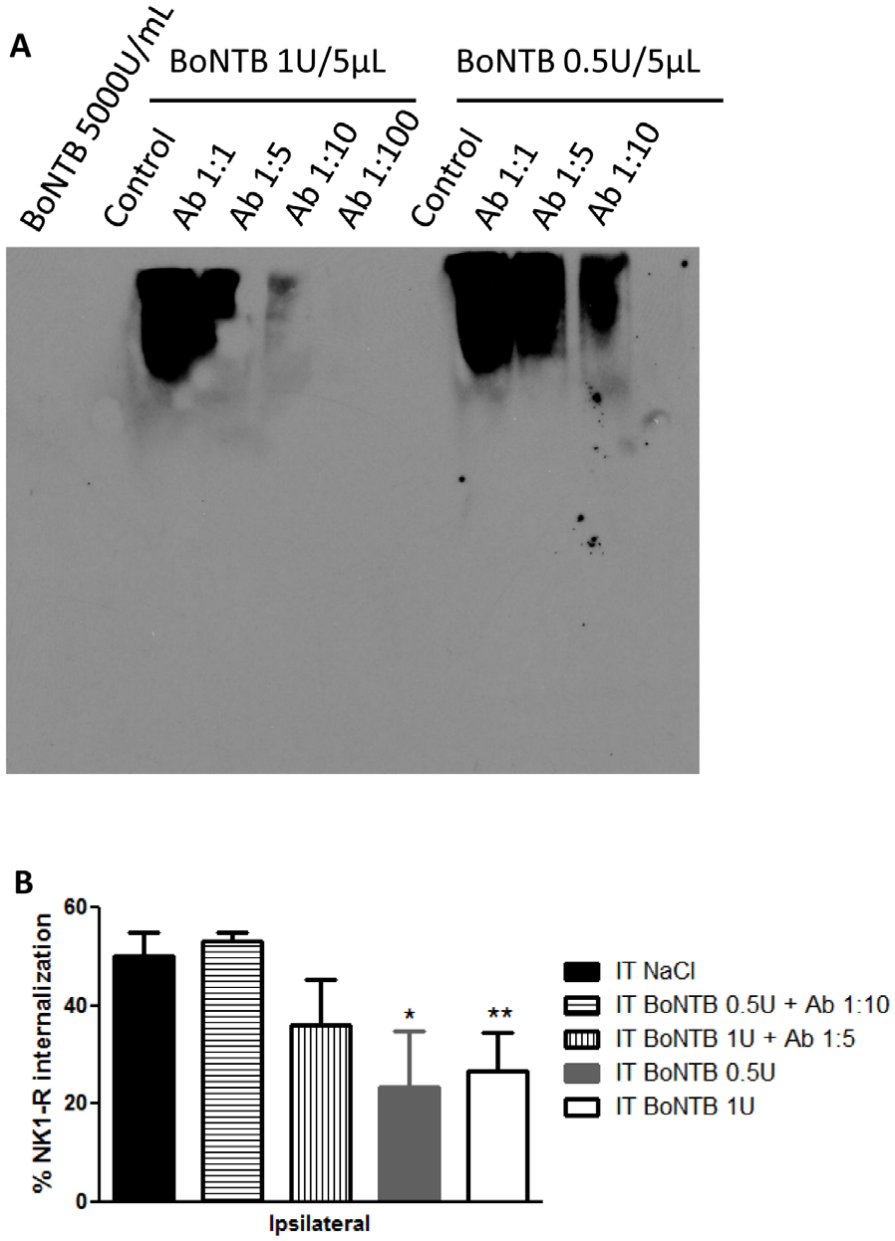
Prevention of BoNT-B effects on primary afferent neurotransmitter release. Effective binding concentrations of BoNT-B and antibody were determined by titer assay on NativePAGE. Co-injection of BoNT-B and anti-BoNTB antibody was administered in mice 2 days prior to IPLT formalin, and spinal cord tissue was harvested at 10 minutes after formalin for analysis of NK1-R internalization. A) Immunoblot of BoNTB-antibody titer assay. BoNT-B was incubated with anti-BoNTB antibody (Ab) at dilutions 1∶1, 1∶5 and 1∶10. The effective binding concentrations were determined to be BoNTB 0.5 U/5 µL incubated with anti-BoNTB antibody at 1∶10 dilution, and BoNTB 1 U/5 µL incubated with antibody at 1∶5 dilution. B) Histogram of NK1-R internalization in the ipsilateral dorsal horn in animals that received IT BoNTB-antibody titer (n = 3), BoNTB 0.5 U–1 U (n = 3), and IT NaCl (n = 4). Bars represent mean ± SEM; P-value<0.05 significance.

Blockade of substance P release from primary afferent C-fiber by intrathecal delivery BoNT-B should required an enzymatically active and mobile neurotoxin. We hypothesized that co-injection of BoNT-B with anti-BoNTB antibody inhibits the effects on neurotransmitter release shown in previous experiments. BoNTB-antibody titer assay showed effective binding concentrations of BoNTB and antibody, as assessed on NativePAGE. BoNT-B concentrations equivalent to IT injection of 0.5 U/5 µL incubated with anti-BoNTB antibody at 1∶10 dilution, and IT BoNT-B 1 U/5 µL incubated with antibody at 1∶5 dilution (figure 8A) resulted in different banding pattern on NativePAGE, as compared to BoNT-B control and BoNT-B with lower antibody concentrations. This suggests difference in protein migration due to difference in charge and/or mass correlated with antibody binding. Mice received IT BoNT-B 0.5 U and 1 U co-injected with antibody at respective titer concentrations 2 days prior to IPLT formalin. Lumbar spinal cord tissue was then analyzed for NK1-R internalization. No statistical difference was shown in NK1-R internalization between mice that received IT NaCl and animals that received IT BoNT-B and antibody co-injection. However, mice that received IT BoNT-B 0.5 U, without antibody, showed reduced NK1-R internalization, as compare to IT NaCl pretreated animals (figure 8B). Thus, incubation with anti-BoNT-B antibody most likely renders the neurotoxin immobile and unable to be internalized across the synaptic terminal membrane.

### Control for target specificity: Resistance to IT BoNT-B in the rat

**Figure 9:**
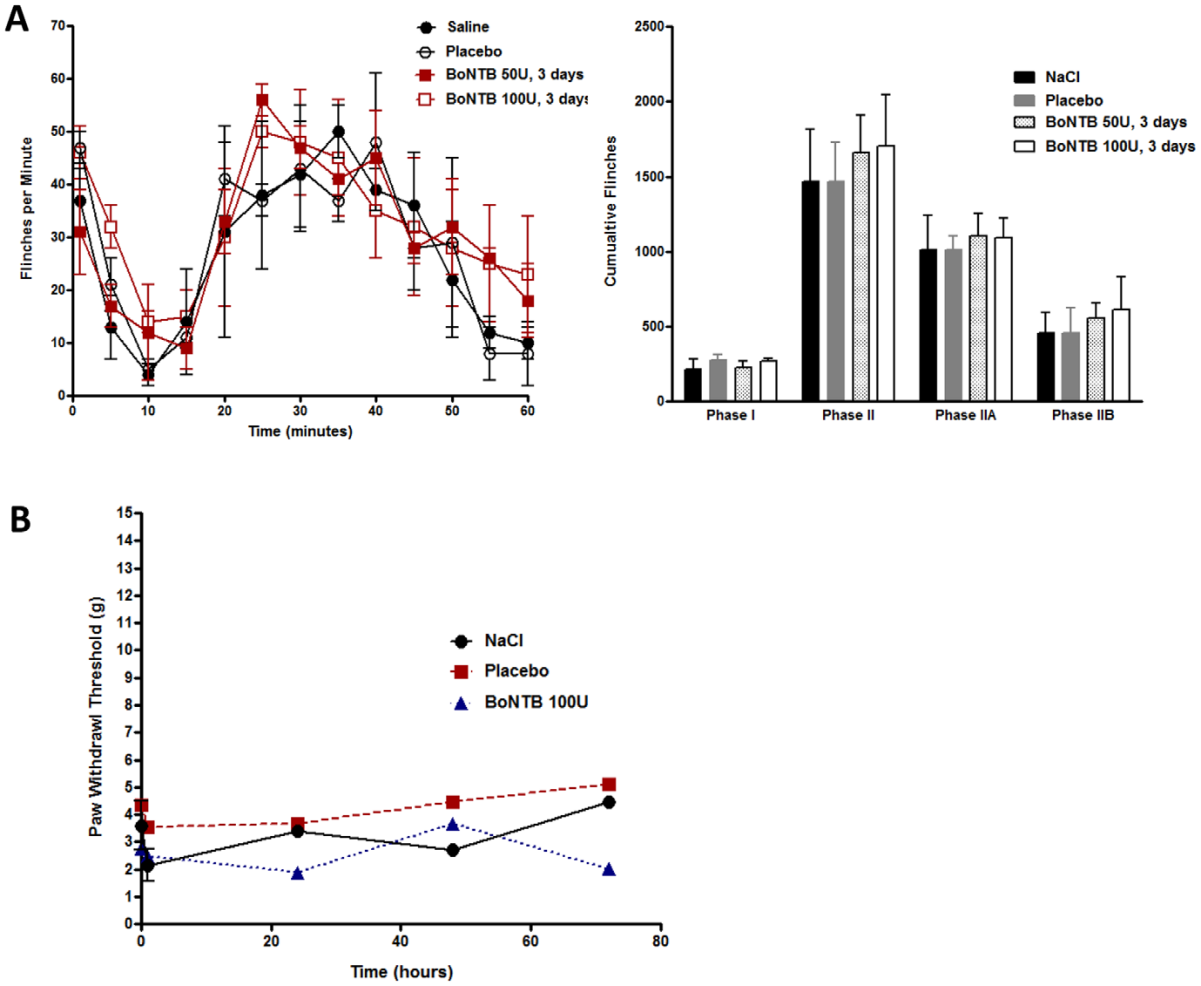
Absence of BoNT-B effect on pain in the rat. A)Time course (left panel) of observed flinching behavior and histogram of phasic cumulative flinches (right panel) comparing phase I (0–10 mins), phase II (10–40 mins), phase IIA (10–20 mins) and phase IIB (20–40 mins). Rats received IT BoNTB (50 U, 10 µL; 100 U, 20 µL; n = 3) 3 days prior to IPLT formalin, as compared to placebo (10 µL; n = 3) and NaCl (10 µL; n = 3) pretreated animals. B) Spinal nerve ligation of the L5 spinal nerve was performed in rats 7–10 days prior to IT injection. On day 0, rats received IT BoNT-B (100 U,20 µL; n = 4), saline (n = 2), or placebo (n = 2). Paw withdrawal threshold in response to von Frey filaments was measured each day for 3 days following IT injections. Bars represent mean ± SEM.

An important question relates to the target specificity of the IT B0NT-B effect. We have hypothesized that these effects of IT BoNT-B in the mouse is the result of BoNT-B specific cleavage of VAMP I/II. In the mouse and human, BoNT-B cleaves VAMP I protein at amino acid residues Gln/76-Phe/77, which results in inhibition of neurotransmitter release. However, rat BoNT-B displays replacement of amino acid residue Val for Gln in VAMP I protein at the cleavage site, rendering VAMP resistant to this cleavage mechanisms [11], [12]. Rats that received IT NaCl or placebo showed typical biphasic flinching behavior evoked by intraplantar formalin. Rats that received IT BoNT-B in doses of 50 U–100 U showed no evident effects upon motor behavior at day 3 when behavior tests were performed and no effects distinct from controls on formalin evoked flinching (figure 9A). In addition, unlike the mouse, IT BoNT-B did not reduce the tactile allodynia in rats after spinal nerve ligation (figure 9B), as compared to allodynic rats that received IT NaCl or IT placebo. This data strongly suggest that BoNT-B specific and active enzymatic cleavage of VAMP I/II is required to produce effects of BoNT-B on neurotransmitter release, nociception, and SNL-induced hyperalgesia in the mouse.

## Discussion

The present *in vivo* studies demonstrate for the first time that the intrathecal delivery of BoNTB produces a prominent block of the evoked release of substance P from small primary afferents and a concurrent effect upon evoked pain behaviors. BoNT-B internalization into spinal cells, its cleavage of VAMP I/II protein, and the lack of BoNT-B effect in the rat with known BoNT-B resistance suggest that intrathecally delivered BoNT-B exerts its effects on neurotransmitter release in part through VAMP I/II cleavage in sensory afferents. Issues pertinent to the interpretations of these findings will be considered below.

### Distribution of intrathecal drugs and the BoNT-B effect profile

Rostrocaudal distribution of the injectate following intrathecal delivery largely depends on volume of the injectate. Five microliters used in this series of studies is the typical volume used in the mouse. Intrathecal delivery of a drug is relatively restricted to the location of injection. In preliminary studies, the intrathecal injection of a blue dye produced distribution up through the mid-thoracic level, which clearly covers spinal segments where primary afferents from the hind paws terminate at the spinal cord. On the other hand, only 3% of intrathecal injectate is found in the brain 10 minutes after intrathecal injection [13]. While this series of studies showed that intrathecal delivery of BoNT-B blocked neurotransmitter release from primary afferent C-fibers and nociception, it is important to appreciate that these effects were not accompanied by motor impairment. Maximal tolerable dose, or LD50, for intracerebroventricular delivery of BoNT-A and BoNT-B has been previously established to be 3.75–15 pg of toxin per mouse of average body weight [14]. The maximal tolerable intrathecal dose reported in our studies is 5 pg per mouse of average body weight, which is within range of toxin concentrations previously described.

We believe that the absence of intrathecal BoNT-B effect on motor function at doses that block spinal neurotransmitter release reflect the spinal localization of BoNT-B effects and lack of penetration of the toxin into deep laminae. Synaptic terminals of small peptidergic primary afferents in the mouse lie in the superficial dorsal horn (laminae I and II), about 25 µm from the surface. Motor neurons lie more deeply (50–100 µ) in the ventral horn. Penetration of the parenchyma after IT delivery depends on concentration gradient, lipid partition coefficient for small molecules, and molecular weight, with large molecules showing a diffusion rate inversely proportional to molecular weight [15], [16]. BoNT-B is a 150 kDa protein. Studies of drug diffusion after intrathecal injection have shown that large molecules diffuse poorly into the parenchyma [17]. Accordingly, superficial localization of BoNT-B results in a relatively restricted distribution of the toxin to the site of injection, which we believe accounts for the potent effect on sensory function and absence of effect on motor function after intrathecal delivery in the mouse at antinociceptive doses. Motor effects were clearly evident after higher doses, a finding that could be consistent with either systemic redistribution or perhaps reuptake and retrograde transport [18].

### Intrathecal BoNTB and substance P release

The principal finding in the present studies is the block of SP release from small peptidergic primary afferents as defined by the reduction in NK1-R internalization. While previous work has shown that BoNT-A inhibits release of acetylcholine, substance P [2], glutamate [3], and calcitonin gene-related peptide [4], [5], we believe these are the first studies to demonstrate an effect upon BoNTB release on peptidergic afferent neurons and to demonstrate this action *in vivo* in a defined neuraxial system. The linkage between NK1-R internalization and afferent SP release is based on several components. 1) There are three sources of SP in the dorsal horn: primary afferents, bulbospinal projections and intrinsic neurons to a lesser extent. Previous work has reported that depletion of SP in bulbospinal pathway has no effect on NK1-R internalization in the superficial dorsal horn, while depletion of primary afferent SP with capsaicin prevents NK1-R internalization otherwise evoked by a noxious stimulus [19]. 2) Intrathecal morphine, which reduces extracellular spinal SP through a pre-synaptic action of peptidergic terminals [20], reduces the fraction of spinal neurons that show NK1-R internalization after stimulation with a noxious stimulus. 3) *In vitro* studies using spinal cord slices have demonstrated covariance between local SP concentration and increase in spinal NK1-R internalization [21]. 4) A variety of stimulus conditions *in vivo* known to initiate activity in peptidergic fibers will increase NK1 internalization in the ipsilateral dorsal horn present [19], [22]. Importantly, while NK1-R expression s consistent along the L1–L5 lumbar segments, NK1-R internalization in the dorsal horn ipsilateral to the site of IPLT formalin injection (and other treatments) is maximal in the ipsilateral L4–L5 segments, consistent with the location of terminals from sensory input originating from the ipsilateral hind paw. Based on these observations, spinal NK1-R internalization is a robust reflection of SP release from spinal primary afferents.

### Role of VAMP cleavage

The mechanisms of action of BoNTs on neurotransmitter release depend on toxin internalization into neurons and the specific cleavage of synaptic proteins. Botulinum neurotoxin structure consists of light chain (LC) - the protease domain responsible for enzymatic cleavage of SNARE proteins, and heavy chain (HC) whose receptor domain which mediates binding to membrane receptor and translocation domain mediates toxin translocation into neurons. Toxin internalization is mediated by LC binding to ganglioside GT1b [23] and synaptotagmin I and II which are transiently expressed on plasma membrane during vesicle recycling [24], as well as contribution of the heavy chain as a conduit for toxin internalization [25], [26]. During neurotransmitter release, Fusion of SNARE proteins syntaxin, synaptobrevin, and SNAP-25, resulting in formation of a four-α-helix bundle – the SNARE protein complex - is critical for vesicle fusion with the plasma membrane and release of neurotransmitters [27]. Enzymatic cleavage of VAMP I/II by LC protease domain of BoNT-B results in failure of formation of SNARE protein complex and prevention of vesicle-membrane fusion during neurotransmitter release.

In the present work, confirmation that BoNT-B effects were indeed the result of VAMPI/II proteolytic cleavage is provided by several experiments. 1) Incubation of tissue lysate with BoNT-B demonstrated a decrease in VAMPI/II protein. 2) Co-injection of BoNT-B with titrated concentrations of anti-BoNTB antibody prevented the *in vivo* effects on formalin-induced NK1-R internalization, in which case the antibody may have rendered the toxin immobile or unable to be internalized into the cytoplasm. 3) Finally, rat resistance to BoNT-B serves as a biological negative control. BoNT-B proteolytically cleaves VAMP I and II at amino acid residues Gln/76-Phe/77 in mouse and human. In the rat VAMP I protein, Gln/76 is replaced by a valine residue, which prevents BoNT-B cleavage [11]. These results jointly suggest that specific VAMPI/II cleavage is required for BoNT-B effect on formalin-evoked neurotransmitter release and nocifensive response.

Recovery from BoNTs has been explored in motor neurons at the neuromuscular junction and has been proposed to require de novo protein synthesis and remodeling at the synapse [28]. Motor neurons have been shown to produce sprouting of processes 32 days after denervation by BoNT-A treatment, where sprouts contain SNARE proteins [29]. Sprouting occurs concurrently with nerve-evoked muscle twitch at 28–30 days after BoNT-A treatment [30]. In the present study, recovery from BoNT-B effect on formalin-evoked C-Fos expression and on nociceptive behavior through 30 days after BoNT-B treatment. This duration is consistent with findings in previous studies using BoNTA through systemic delivery. Based on the similar properties between BoNT-A and BoNT-B, it is reasonable to believe that similar mechanisms underlie recovery from intrathecal BoNT-B effects shown in the present study.

### Effects of Intrathecal BoNT-B on spinal nociceptive processing

IT BoNTB, at doses having no effect upon motor function produced persistent (as long as 30 days) and significant effects upon the phase 2 of the formalin-induced flinching response and reversed the tactile allodynia otherwise occurring after nerve injury for at least 15 days. Importantly, the pharmacology of these various behavioral states is distinct. The formalin model, the biphasic flinching behavior is believed to reflect the acute afferent excitation during the phase 1 and an ongoing low level of afferent traffic during the phase 2 of the behavioral response which evokes prominent flinching as a result of potent facilitated state initiated by the high level of afferent input observed during phase 1 [31], [32]. Nerve injury leads to a persistent tactile allodynia. Several mechanisms are considered to underlie this facilitated state, including dorsal horn reorganization, a loss of inhibitory control, a corresponding increase in dorsal horn excitability [33], [34] and associated with an increase in spinal glutamate release [35].

Phase 2 flinching, but not phase 1 flinching, and post nerve injury tactile allodynia are blocked by spinal NK1-R receptor antagonists [36], [37], [38]. The failure to observe effects of NK1-R blockade in nerve injury pain is consistent with the lack of evoked release of SP by tactile stimulation in nerve injured animals [39]. It should be stressed that other spinal transmitter systems, notably those for glutamate released form primary afferents and from dorsal horn excitatory interneurons, are known to play a critical role in both phases of the formalin model and the post nerve injury allodynia [40], [41], [42]. IT delivery of AMPA antagonists will block phase 1 and phase 2 formalin as well as tactile allodynia, whereas NMDA antagonists will reduce phase 2 flinching only and nerve injury evoked tactile allodynia [31], [43], [44]. Consistent with the ability of BoNTB to produce a persistent block of excitatory drive in the spinal dorsal horn, we noted that the increase in C-Fos expression following intraplantar formalin [45] and was persistently blocked by IT BoNT-B at doses which attenuated the evoked SP release and phase 2 formalin evoked flinching and the nerve injury evoked tactile allodynia.

It is evident that IT BoNT-B will attenuate SP release, but we believe that functional profile outlined above reflects on a more complicated action. As noted, if the effects of IT BoNT B were uniformly expressed on all releasing functions of the peptidergic neurons, then we would expect that there would be a potent effect upon the release of other transmitters, notably glutamate. Had glutamate release from small afferents been blocked, we would expect to see a loss of phase 1 flinching, which was not observed. Moreover, if block of the afferent SP release were the only point of interaction, we would expect that the recovery from BoNT-B effects on all measures to be comparable. As noted, SP release recovered between 5 and 15 days post injection, whereas the block of the formalin-evoked flinching was comparable to the continued suppression of C-Fos, persisted for 30 days. Similarly, the pharmacology of the anti-allodynic effect in nerve injury suggests a role for glutamate, but fails to support a role for SP. In any case, the block of allodynia persisted robustly for at least 15 days, beyond the time of recovery for the SP release block. We thus conclude that the IT BoNT-B was exerting an effect not only on SP release but on that of other spinal transmitters, notably glutamate, and not only on primary afferent terminals, but those perhaps from inter-neurons. Previous work has shown that BoNTs block glutamate release from motor neurons and hippocampal neurons [3]; these effects are also mediated by cleavage of SNARE proteins [46]. It is important to emphasize that the observed changes in sensory function after IT-BoNT-B did not generally alter ambulation or normal function which would have occurred had there been a promiscuous block of all large afferent transmission (e.g. a phenomena commonly observed with various ionotrophic glutamate receptor antagonists). An additional possibility is suggested by the observation that BoNT-B can inhibit long term potentiation (LTP) in hippocampal CA1 pyramidal cells without blocking baseline synaptic responses [47], [48]. LTP is considered to have mechanistic similarities to spinal facilitated states as manifested in the formalin model. Failure to see an effect upon baseline responses in hippocampal systems parallels our observation that IT BoNT-B had no effect upon phase 1 formalin or acute nociception, but diminished the facilitated state. Of interest, the effects of BoNT-B on LTP were believed to be associated with a suppression of trafficking of AMPA receptors into the membrane [48]. It will be interesting to determine whether a similar phenomenon applies to the present observations with intrathecal BoNT-B.

In summary, the present studies emphasize the potent effect of BoNT-B on the *in vivo* release from a well-defined pool of peptidergic primary afferents. In addition, given the differential pharmacology and recovery intervals for the several effects examined, we believe these studies emphasize the likely effects on the release of other spinal transmitters and upon non-afferent terminals. It also raises the question as to the potential differential effects exerted by BoNT serotypes and their derivatives. Importantly, the *in vivo* model allows definition of the behavioral consequence of the spinal drug action and permits demonstration of an effective therapeutic rationale which likely reflects the access of the intrathecal drug to deeper neurons. These studies thus suggest a potential therapeutic direction for a persistent single dose regulation of spinal nociceptive processing. It should be emphasized that the use of such persistent agents into the human spinal canal must be subject to extensive preclinical safety evaluation [49], [50].
