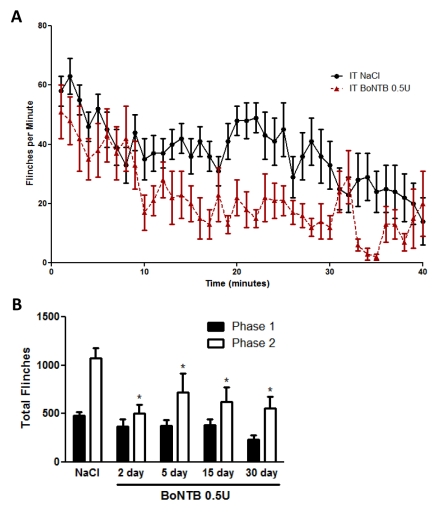# Correction: Spinal Botulinum Neurotoxin B: Effects on Afferent Transmitter Release and Nociceptive Processing

**DOI:** 10.1371/annotation/1fe31fa2-e930-4a72-a208-64eb42d99f02

**Published:** 2011-08-22

**Authors:** Polly P. Huang, Imran Khan, Mohammed S. A. Suhail, Shelle Malkmus, Tony L. Yaksh

There are typographical errors in Figure 6. The correct Figure 6 can be viewed here: 

**Figure:**