# Correction: Spinal Botulinum Neurotoxin B: Effects on Afferent Transmitter Release and Nociceptive Processing

**DOI:** 10.1371/annotation/7b989904-0cf4-4f2d-9626-6bf3ef6646c5

**Published:** 2011-08-22

**Authors:** Polly P. Huang, Imran Khan, Mohammed S. A. Suhail, Shelle Malkmus, Tony L. Yaksh

The p value in the legends of Figures 3, 4, 5, 6, and 7 are incorrect. The correct p values are: "< 0.05."

